# Diagnostic challenges in systemic amyloidosis: a case report with clinical and laboratorial pitfalls

**DOI:** 10.4322/acr.2021.326

**Published:** 2021-01-10

**Authors:** Angelina Maria Martins Lino, Jussara Bianchi Castelli, Roberta Shcolnik Szor, Fabio Fernandes, Vera Demarchi Aiello

**Affiliations:** 1 Universidade de São Paulo (USP), Hospital das Clínicas, Department of Neurology, Clinical Peripheral Nerve Group, São Paulo, SP, Brasil; 2 Universidade de São Paulo (USP), Instituto do Coração, Laboratory of Pathology, São Paulo, SP, Brasil; 3 Grupo Fleury, Department of Pathology, São Paulo, SP, Brasil; 4 Universidade de São Paulo (USP), Instituto do Câncer do Estado de São Paulo, São Paulo, SP, Brasil; 5 Universidade de São Paulo (USP), Instituto do Coração, Cardiomiopathy Group, São Paulo, SP, Brasil

**Keywords:** Polyradiculoneuropathy, Chronic Inflammatory Demyelinating, Diagnostic Errors, Amyloidosis, Familial, Light Chain Immunoglobulin Amyloidosis, Paraproteinemias

## Abstract

Currently, there is growing evidence in the literature warning of misdiagnosis involving amyloidosis and chronic inflammatory demyelinating polyneuropathy (CIDP). Although inducing clinical manifestations outside the peripheral nervous system, light chain and transthyretin amyloidosis may initially present with peripheral neuropathy, which can be indistinguishable from CIDP, leading to a delay in the correct diagnosis. Besides, the precise identification of the amyloid subtype is often challenging. This case report exemplifies clinical and laboratory pitfalls in diagnosing amyloidosis and subtyping amyloid, exposing the patient to potentially harmful procedures.

## INTRODUCTION

Misdiagnosis is a well-known problem in medical practice, delaying the correct diagnosis and introducing the best therapeutic approach. In Neurology practice, peripheral neuropathy (PN) may present with varied clinical phenotypes, with or without associated systemic diseases, easily inducing misdiagnosis. In this setting, amyloidosis and the clinical spectrum of CIDP are examples.

The agreement rate among experts for typical and atypical CIDP is 97% and 60%, respectively, and initial alternative diagnosis is more likely in atypical CIDP due to the concomitance of systemic diseases.^1^ Atypical CIDP is a heterogeneous group in which distal acquired demyelinating symmetric (DADS) neuropathy accounts for 5-13% of cases, presenting with sensory ataxia, distal motor involvement and fulfilling demyelination criteria of the European Federation of Neurological Societies and the Peripheral Nerve Society (EFNS/PNS).^2^^-^5 Having refractoriness to immunotherapy and preferential involvement of men from the 6^th^ to the 8^th^ decade of life, monoclonal gammopathy of undetermined significance (MGUS) is found in two-thirds patients with DADS, 60% associated with immunoglobulin (Ig) M, 30% IgG, and 10% IgA.^6^^,^^7^ MGUS can progress to light-chain amyloidosis (AL), representing a differential diagnosis in a previously diagnosed MGUS-associated CIDP.

Likewise, amyloidosis is a heterogeneous group in which different patterns of PN can be observed. In AL, PN may be present at the disease onset in up to 20% of cases and represent the main clinical feature in some inherited variants of transthyretin (TTR) amyloidosis (ATTRv).^8^^,^^9^ The clinical overlapping between AL and late-onset ATTRv may hinder the recognition of the amyloid subtype.

Since AL is the most common subtype of systemic amyloidosis with incidence up to 10 cases/million/year, hereditary forms might be misdiagnosed as AL due to absence of familial history, same age range, the common finding of MGUS in the elderly, or false-positive immunohistochemistry staining.^8^^,^^10^^,^^11^ In a series of 350 presumptive AL cases, 9.7% harbored a hereditary cause, being TTR mutation found in 38% of them.^12^ TTR variants are the worldwide commonest cause of hereditary systemic amyloidosis, in which the nervous system and heart involvement may be indistinguishable from those in AL. In the late-onset ATTRv, presenting symptoms occurs in (i) patients over 50 years, (ii) with male predominance, (iii) not striking family history due to low gene penetrance rate, (iv) loss of all sensory modalities rather than unmyelinated fibers at beginning, (v) relatively mild autonomic dysfunction, and (vi) more organ involvement (heart, kidney, eye).^9^^,^^11^^,^^13^

The most common TTR mutation, Methionine for Valine at position 30 (Val30Met), shows clinical and electrophysiological heterogeneity, depending on the (i) patient’s geographical origin (endemic versus non-endemic area), (ii) ethnic group, (iii) incomplete penetration of gene mutation, and (iv) age of disease onset.^9^^,^^11^^,^^13^ Misdiagnosis of ATTRv as CIDP ranges from 20 to 61%.^10^^,^^14^ Adding challenges to diagnosis, cytoalbuminologic dissociation in the cerebral spinal fluid (CSF) and electroneuromyographic (ENMG) features obeying demyelinating criteria for CIDP were found in 23% and 39%, respectively, in symmetrical and asymmetrical neuropathies in late-onset ATTRv.^14^^-^17

Herein, a clinical case exemplifies the diagnostic challenges in systemic amyloidosis.

## CASE REPORT

A previously healthy 67-year-old Caucasian male was admitted to the outpatient service of Neurology Division in April 2015, complaining of numbness of hands and feet, and inability to walk that had started two years before with progressive worsening, associated with weight loss of 14kg. He denied dry mouth and eyes or sweating, gastrointestinal or sexual dysfunction, previous diseases, and any relevant familial medical history. No abnormalities were found on general clinical examination, but skinny complexion. At neurological assessment, Medical Research Council scale (MRC) was grade 5 in all muscles, except grade 1 for flexion and extension of the feet; slight atrophy was found in feet intrinsic muscle added to global areflexia, positive Romberg sign, superficial hypoesthesia at toes, severe loss of vibratory sensation up to elbows and knees. Blood pressure and heart rate were 130x82 mmHg and 83 bpm, respectively, on supine position, and 125x80 mmHg and 92 bpm, respectively, after 3 minutes on standing position; cranial nerves were preserved; 51 points on Neuropathy Impairment Score (NIS); score 2 for upper (U) and 2 for lower (L) limbs (L) on Overall Neurological Limitation Scale (ONLS). Except for IgA lambda-MG in serum immunofixation, a slightly elevated serum beta-2 microglobulin of 2.0 mg/mL (RR = 1.0-1.7 mg/mL) and mild anemia (hemoglobin of 11,5 g/dL, RR = 13-18 g/dL), other laboratory tests were normal, including complete blood count, vitamin B12 and folic acid serum levels, serum kappa and lambda free light-chains ratio, liver, renal and thyroid functions, blood glucose, lactic dehydrogenase, calcium, serology for infectious diseases (HIV, B, and C hepatitis, VDRL), antinuclear and rheumatoid factors, antineutrophil cytoplasmic antibodies, anti-SSA/SSB, total complement and fractions, anti-cardiolipin, prostatic specific antigen, carcinoembryonic antigen, alpha fetus protein, and urinalysis with protein electrophoresis and immunofixation. Computed tomography (CT) of chest, abdomen, and pelvis were normal, and ^99m^Tc-MDP scintigraphy (SC) did not show abnormal uptake in bones. Bone marrow aspirate was normal. The CSF showed 1 cell/mm^3^ and 88 mg/dL (RR < 45mg/dL) of protein with 16% gamma (RR < 14%). Anti-nerve antibodies detection was not available. An external ENMG showed longer motor distal and F wave latencies, moderately reduced motor nerve conduction velocity, no conduction block or abnormal temporal dispersion, slightly reduced motor amplitude data, and was interpreted as a primarily demyelinating process with slight secondary axonal degeneration fulfilling demyelination criteria of EFNS/PNS.^2^

DADS with IgA lambda-MGUS was diagnosed, and the patient started on monthly pulse therapy with intravenous (iv) methylprednisolone (MP, 1000 mg/day/3days) from May to October 2015. No clinical-laboratory evidence of any systemic disease arose, NIS remained unchanged, but worsening in ONLS (2 UL and 3 LL) lead to the addition of monthly cyclophosphamide (1g/iv/month) to MP from December 2015 to May 2016, and then changed to oral methotrexate (15 mg/week) for 7 months.

In March 2016, under immunosuppressive therapy, the patient started with sexual distress complaints. Except for the IgA lambda-MG, extensive serum reinvestigation and thoracic and abdominal CT remained normal. The ENMG (Table 1) was remade and showed unexpected and marked axonal impairment.

**Table 1:** Nerve conduction study (2016)

Nerve	Motor				Sensory	
	CMAP (mV) L/R	CV (m/s) L/R	L (ms) L/R	FWL (ms) L/R	SNAP (µV) L/R	CV (m/s) L/R
Median	1.2/1.7	34.1/33.5	5.5/5.8	ND/ND	U/U	U/U
Ulnar	1.7/2.3	32.5/35.1	4.7/4.8	52.2/47.8	U/U	U/U
Peroneal	U/U	U/	U/U			
Tibial	U/U	U/	U/U			
Femoral	6.0/4.6	5.6/5.8				
Sural					U/U	U/U

CMAP: compound muscle action potential; CV: conduction velocity; L: distal latency; FWL: F-wave latency; L: latency; m/s: meter per second; ms: millisecond; mV: milli Volt; µV: microVolt; ND: not done; SNAP: sensory nerve action potential; U: unexcitable nerve

**Figure 1:**
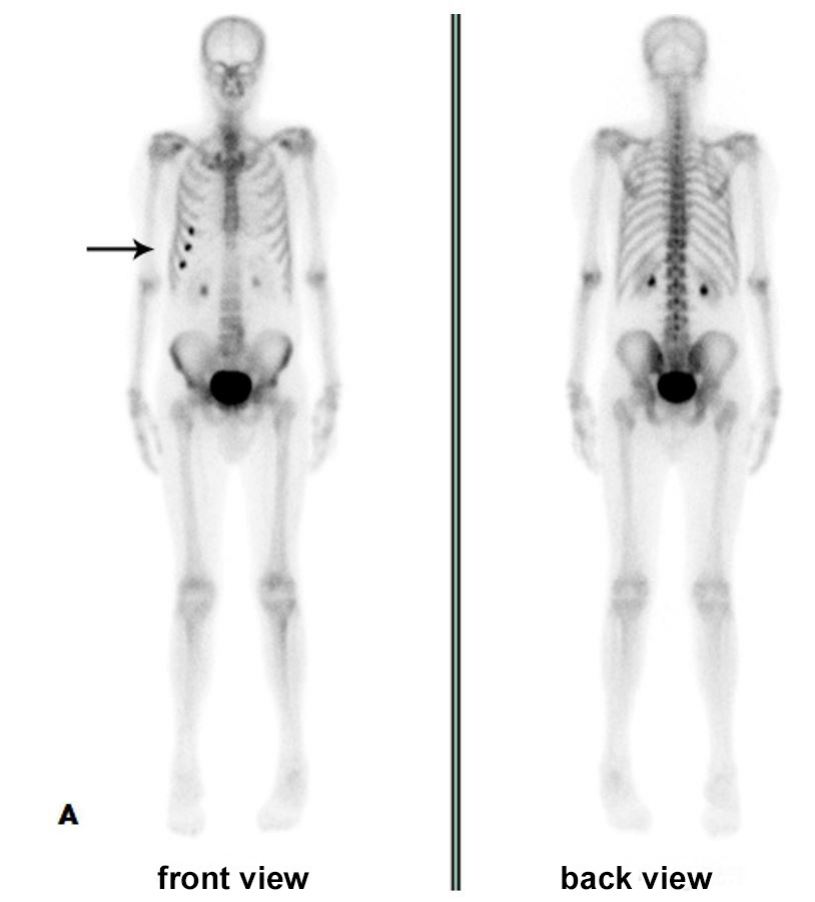
Bone scintigraphy with ^99m^Tc-MDP in May 2016 showing abnormal bone uptake in 5^th^, 6^th^ and 7^th^ right coastal arches (arrow).

**Figure 2:**
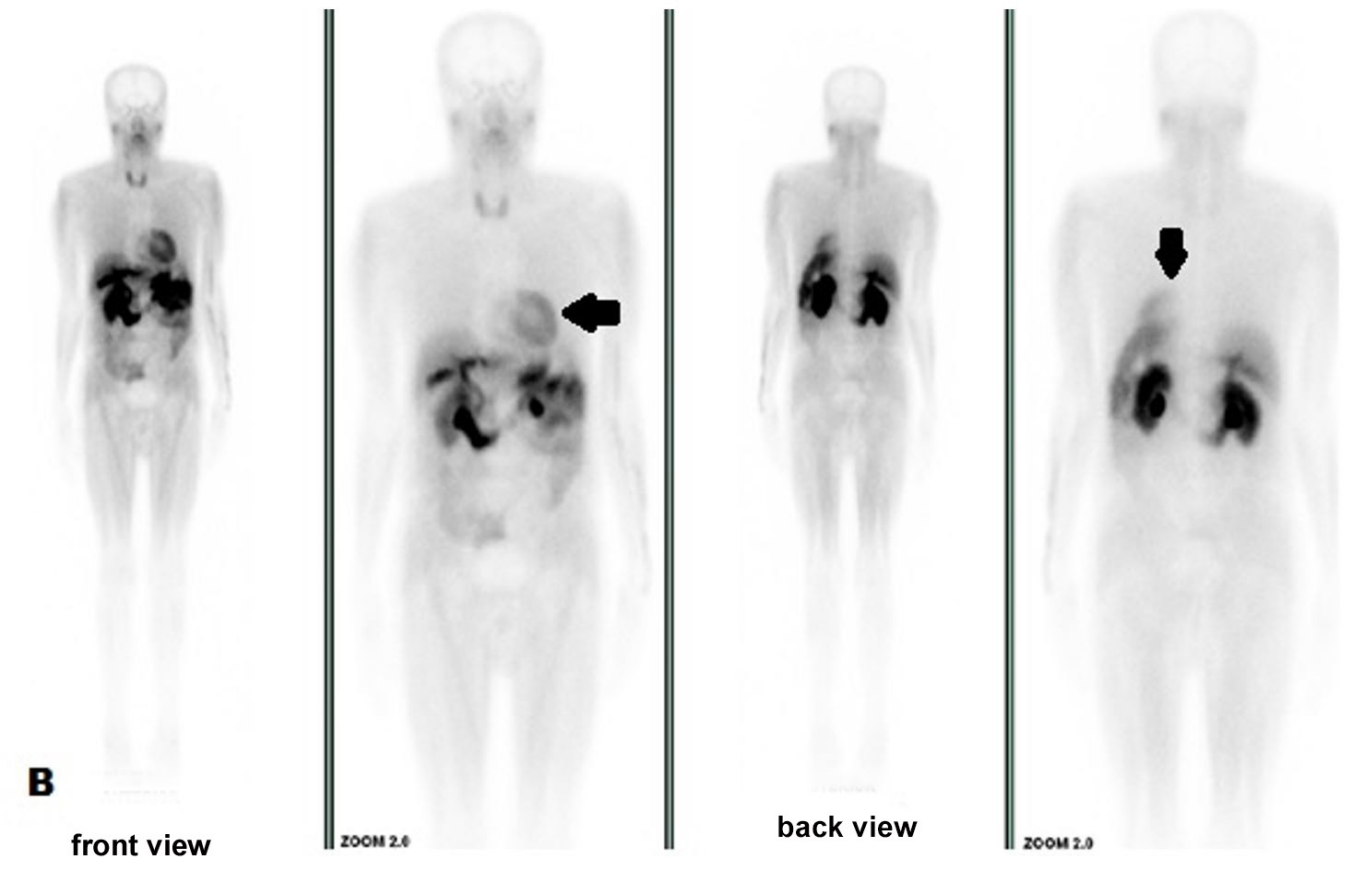
Normal bone uptake with slight uptake in the myocardium (arrow) at bone scintigraphy with ^99m^Tc-MIBI in November 2016.

Autonomic evaluation disclosed the absence of cutaneous sympathetic reflex in all limbs and reduced cardiac variability at rest and with a deep breath and Valsalva maneuver (RR interval = 901.2 ± 4.6 ms). The uptake in fifth and seventh ribs observed in ^99m^TC-MDP SC (Figure 1) was not confirmed in ^99m^TC-MIBI SC (Figure 2). No plasmacytosis or other alterations were seen in bone marrow aspirate, and a subcutaneous (SC) tissue biopsy resulted negative for amyloid deposition under Congo red staining.

**Figure 3:**
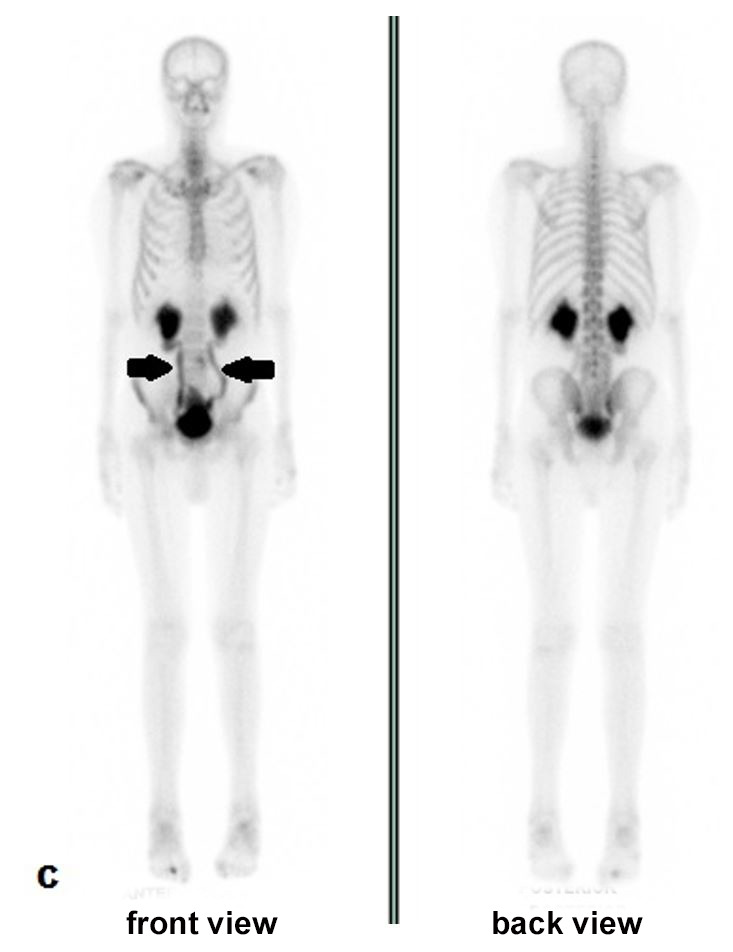
Normal bone uptake with ^99m^Tc-MDP in October 2017, showing distension of urinary tract (arrows).

Despite the negative SC biopsy result, AL was still suspected associated with the IgA lambda component. Due to the marked motor worsening (NIS 88.5, ONLS 3 UL and 5 LL) and the onset of pain and other autonomic complaints (orthostatic intolerance and voiding dysfunction), Fludarabine (100 mg/iv/monthly) was prescribed from December 2016 to April 2017, resulting in slight improvement (NIS 83.5, ONLS 3 UL and 4 LL) and disappearance of bone uptake in coastal arches but showing distension of urinary tract (Figure 3).

**Figure 4:**
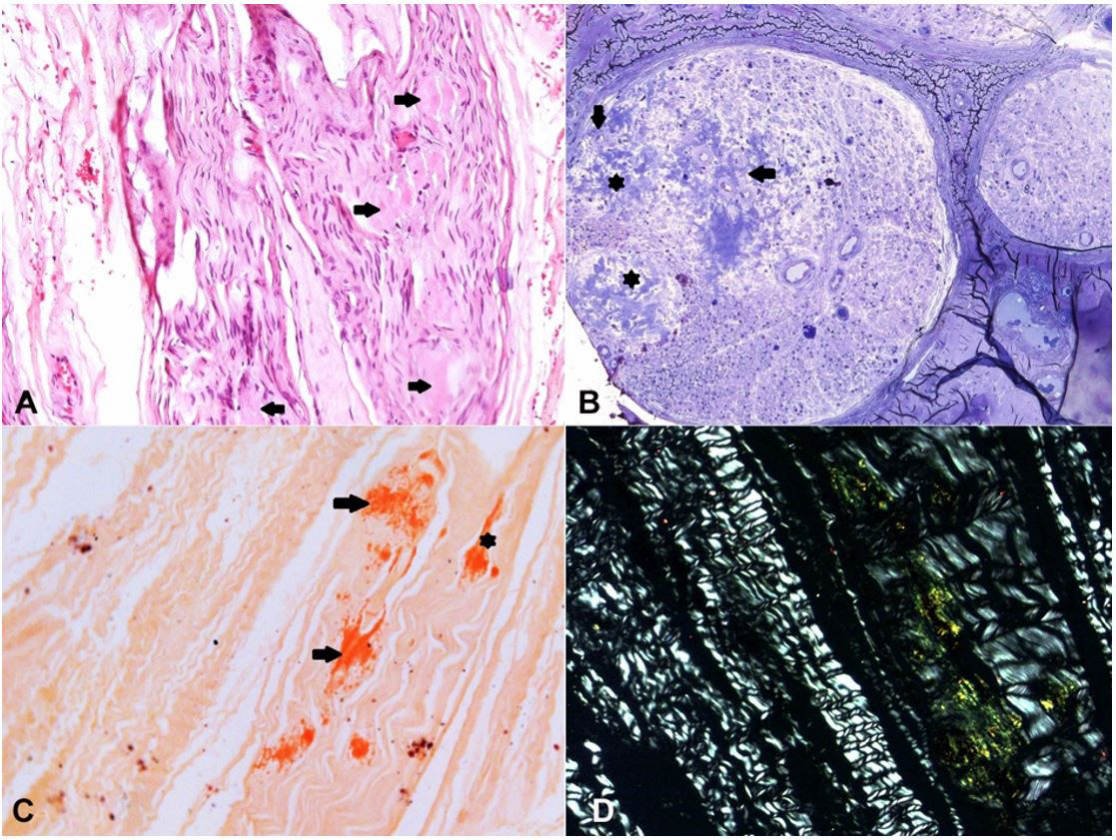
Optic microscopy of sural nerve biopsy. **A** – Extracellular eosinophilic deposits in the endoneuro (arrows) (H&E, X200); **B** – Semithin section (araldite-embedded) showing nerve fascicles with severe loss of thick and thin myelinated fibers and large amyloid deposits in the endoneuro (asterisk) and around endoneural blood vessels (arrows) (Toluidine blue, X200); **C** – Congo red staining showing deposits in the endoneuro (arrows) and epineuro (arrowhead) (X100) showing endoneural apple-green birefringence under polarized light in **D** (X200).

**Figure 5:**
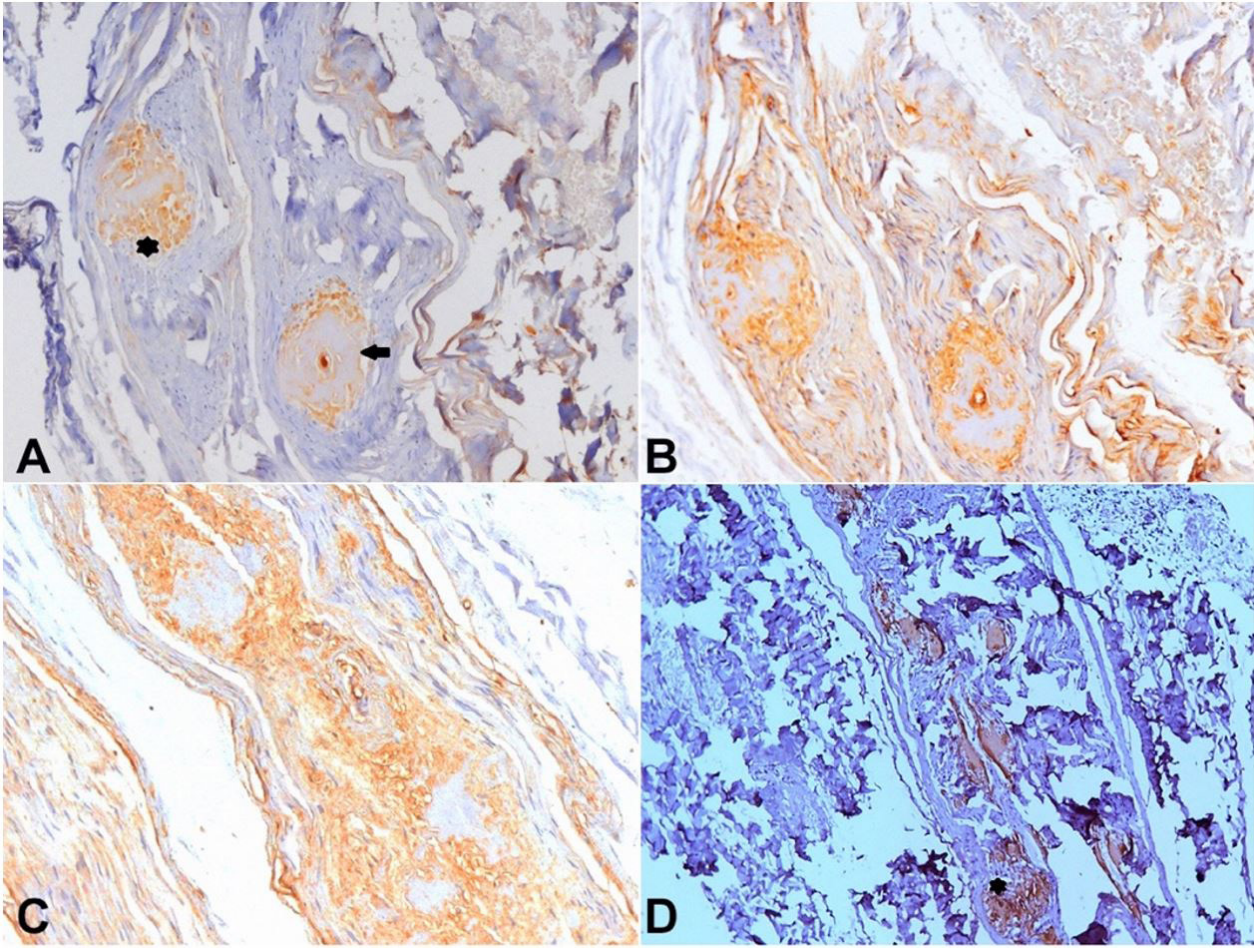
Brown immunolabeling with anti-IgA antibody (**A**) showing two large positive areas in the endoneuro (asterisk) and around an epineural blood vessel (arrow). Intense non-specific background immunolabeling was seen with anti-lambda (**B**) and anti-kappa (**C**) light-chains antibodies [Horseradish peroxidase, X200]. **D** – Brown immunolabeling with anti-Transthyretin antibody showing large positive amyloid deposits in the epineuro at the same endoneural area showed in 5A (asterisk) [Horseradish peroxidase, X100].

A sural nerve biopsy (February 2018) revealed amyloid deposition (Figures 4) with IgA positivity and non-specific lambda light-chain staining by immunohistochemistry (IHC) (Figures 5A-C). Kidney dysfunction started in 2018 with a protein/creatinine ratio of 1,59 (RR = 0.06 – 0.35). Imaging studies showed renal parenchyma with normal thickness and echotexture associated with dilatation of urinary tract and thickened and trabeculated bladder walls (data not shown).

Still denying any suspected familial history, the patient brought us in October 2018 a photograph of his mother at the age of 60 years old showing a very lean lady with atrophic hands, who was bedridden for several years and died at 82 years old due to a non-confirmed bone malignancy. This information led us to search for a mutation in the *TTR* gene to find a heterozygous Val30Met mutation. At the age of 69 years, a liver transplant was not indicated due to the risk associated with age, low body mass index, and renal dysfunction. Tafamidis was also not indicated due to familial amyloidosis staging system score of II (ambulatory but require assistance) and IIIb at polyneuropathy disability score (walking with the help of two sticks). Only in 2019 was it possible to carry out an IHC staining for TTR in the stored nerve tissue biopsy, which showed positive staining at the same sites of previous IgA deposition (Figure 5D).

**Figure 6:**
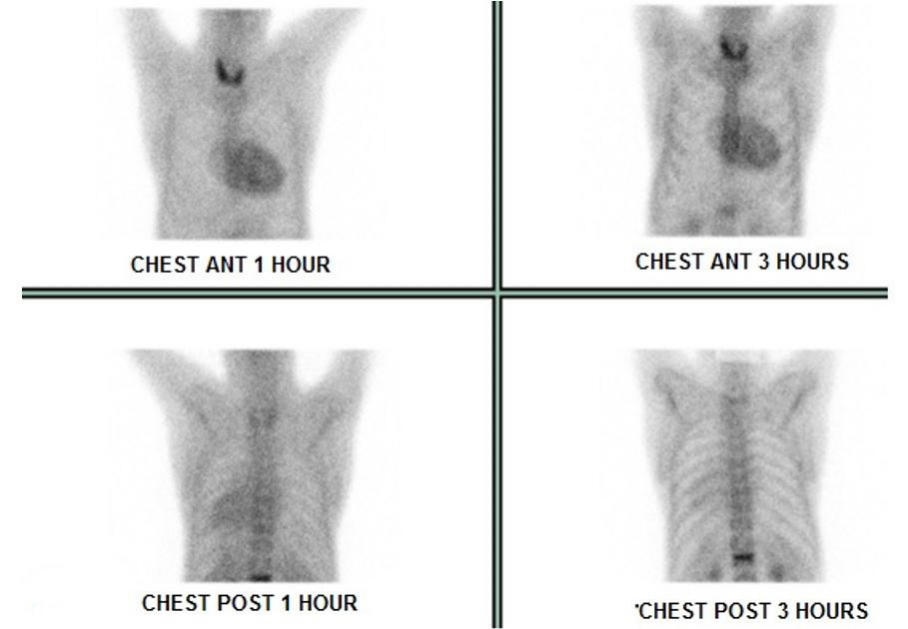
Strong diffuse uptake in ventricular walls at 99mTc-pyrophosphate scintigraphy in May 2019.

Tc^99m^-pyrophosphate SC showed strong diffuse uptake in ventricular walls in February 2019 (Figure 6), with heart/contralateral area ratio of 1.9 and 1.7 after 1 and 3 hours, respectively, highly suggestive of ATTR. At the last follow-up in April 2019, the patient reported a stable neurological condition, sporadic pain in lower limbs, and denied any autonomic symptoms; NIS and ONLS had not changed since 2017. The patient deceased in July 2019 of undetermined cause in another medical service where the autopsy was not performed.

**Figure 7:**
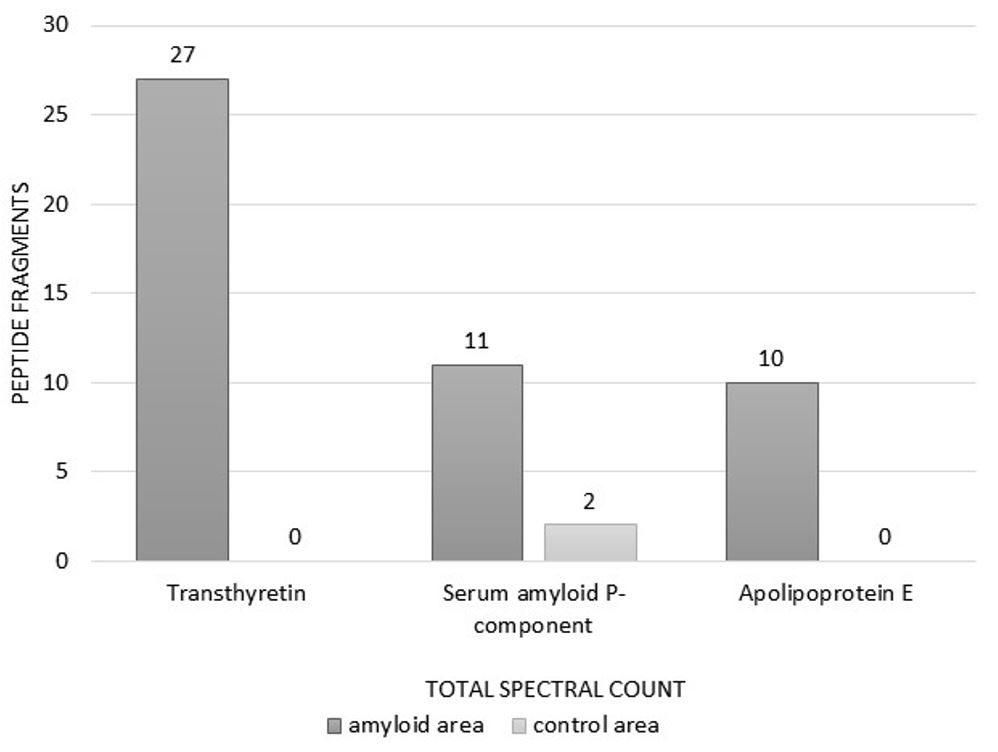
Mass spectrometry data of the sural nerve. After selecting the amyloid deposit area, tissue microdissection and laser capture, the analysis of the peptide fragments revealed the presence of three proteins: transthyretin as the amyloidogenic protein and two other constituents common to all types of amyloidosis, apolipoprotein E and serum amyloid P-component. The protein recognition occurs when 3 or more peptide fragments of a specific protein are identified in the sample [spectra acquired by Q-Exactive HF-X spectrometer and analised using MaxQuant, version 1.6.15.0].

Being performed in January 2021, under research settings, laser microdissection and mass spectrometry analysis (LC/MS) showed only TTR in amyloid on nerve biopsy (Figure 7).

## DISCUSSION

This case report exemplifies some challenging aspects that lead to misdiagnosis of systemic amyloidosis in a patient who consecutively received a diagnosis of DADS, AL, and finally ATTRv.

Early and severe sensory ataxia, dissociated CSF, findings of demyelination in the first ENMG, presence of MG, the initial absence not only of autonomic symptoms but also of positive family history, and an IHC staining showing two amyloid types in nerve biopsy were the main pitfalls in our patient.

Amyloidosis should be hypothesized as a cause of a PN when early marked symptoms and signs of autonomic dysfunction and/or severe neuropathic pain with a length-dependent pattern are inaugural manifestations or appear during the clinical course in a neuropathy initially attributed to another cause.^9^^,^^11^^,^^13^^,^^18^ In our case, the emergence of marked autonomic dysfunction only after two years of follow-up worked as a red flag that drove the clinical reasoning for amyloid deposition, initially thought associated with IgA lambda-MG, later confirmed due to mutated TTR.

Other important clues for amyloidosis are a combination of hereditary neuropathy with general multi-symptom involvement or, in sporadic patients, combinations of PN with cardiac manifestations or severe and progressive carpal tunnel syndrome (CTS) in association with other systemic manifestations.^9^^-^11 Often bilateral, CTS can predate other symptoms in ATTRv and AL by a mean period of 4-6 years and 1 year, respectively.^3^^,^^18^ Having overlapping clinical manifestations, the cardiovascular, gastrointestinal, and genitourinary autonomic system are affected in up to 65% of patients with AL and 75% of patients with ATRRv.^18^

Considering the whole-body nuclear imaging with bone tracers, amyloidosis can be suspected when there is uptake in unexpected areas, particularly in the heart. Currently, ^99m^Tc-PYP has been used in distinguishing ATTR from AL in advanced cardiac amyloidosis, as in our patient. In the absence of MG, a positive cardiac ^99m^Tc-PYP shows 99% sensitivity and 86% specificity but lacks specificity for ATTR in patients who have concomitant MG.^19^ Other ^99m^Tc-labelled phosphate derivatives, such as ^99m^TC-DPD and ^99m^Tc-HMDP, can be uptake due to high calcium compounds in the amyloid with variable sensitivity.^20^^,^^21^ Despite being criticized in searching multiple myeloma lesions, as used in our case, ^99m^Tc-MIBI imaging normally shows relatively high uptake in normal metabolically active tissues, such as myocardium and has no utility in cardiac amyloid detection.^22^^-^26 Tracing sympathetic innervation, ^123^I-MIBGF seems to be useful for early diagnosis in cardiac amyloidosis, specifically in ATTRv.^20^^,^^21^ ^99m^Tc-Aprotinin and ^123^I-SAP are potential amyloid tracers, but do not make a distinction between the different amyloid subtypes.^20^^,^^21^ Conventional cardiac magnetic resonance imaging is most helpful in establishing or confirming the diagnosis in later stages.^19^^,^^26^ However, functional assessment with mitral inflow peak velocities, deceleration times, and other parameters can assist in early diagnosis. Also, atrial gadolinium uptake along with abnormal gadolinium kinetics should make aware of cardiac amyloidosis.^26^

An important pitfall was the initial absence of familial history in our case. In ATTRv, the absence of family history varies from 33% to 100% and is more frequently observed in late-onset patients from non-endemic areas.^9^^,^^12^^,^^14^^,^^27^ However, when a relevant family history exists in patients with PN with prominent autonomic and/or painful symptoms, hereditary causes of systemic amyloidosis should be considered. With autosomal dominant-inheritance in all, the three more important proteins associated with familial amyloid polyneuropathy are TTR, by far the most frequent, apolypoprotein AI, and gelsolin.^10^^,^^19^

The distinction between causal factors and incidental findings in a patient with PN and M protein is difficult. The prevalence of MG in all cases of PN ranges from 3 to 5% and can reach up to 10% in neuropathies with no identifiable cause.^6^^,^^19^^,^^28^ Moreover, the rate of 1% per year of progression to hematologic malignancies, all forms of MGUS can progress to AL or cause organ damage due to immunogenicity of M protein, being the peripheral nerve one of the targets. MGUS is associated with a higher relative risk of CIDP and autonomic neuropathy, 5.9 and 3.2, respectively.^28^ Concurrent presence of MG associated with misdiagnosis in ATTR patients ranges from 12% to 49%, being essential to obtain biopsies from different sites and organs to proceed the adequate amyloid typing.^14^^,^^18^

Data regarding two types of amyloid coexisting in the same patient is scarce in the literature. Koba et al.^29^ reported the IHC findings of Ig (kappa and lambda)-derived and keratin-derived amyloid in one patient with basal cell carcinoma and no MG or Bence-Jones protein. Using immunogold labeling technique and detailing hematological data, Martini et al.^30^ showed tissue simultaneity of two amyloid types in four patients with MG, wild type (wt) ATTR and AL in two cases, AL and SAA in one, and ATTRwt and SAA in another patient.

Diagnosing two amyloid types at different times, Jhaveri et al.^31^ reported two patients with confirmed bone marrow AL by IHC in whom only cardiac ATTRwt was found by LD/MS technique after 6 and 20 years of complete hematological response. Mahmood et al.^32^ showed distinct positive amyloid areas to TTR or light-chain in the myocardium biopsy by IHC, in which LD/MD detected both amyloidogenic precursors in all deposits, but in distinct proportions.

In a retrospective review from the Mayo Clinic, only nine in 1904 patients with LD/MS-proven amyloid in different tissues were identified as having two different amyloid types.^33^ All nine cases showed ATTR as one of the amyloid types, being mutated TTR in only one. The second type of amyloid was Ig-derived in seven, SAA and insulin-derived in one patient each. Regarding tissue distribution, two separated anatomical sites were identified in four patients and in the same tissue sample in five.^33^

Revising our case after LD/MS that only identified ATTR in nerve tissue, the peripheral nerve lesion was imputed to ATTRv, with a false-positive IHC reaction to AL due to concomitant IgA lambda-MGUS. However, considering that an autopsy was not performed, AL in other target organs cannot be ruled out.

Recognition of amyloidogenic protein is a critical step for therapeutic decisions. Although IHC is a validated tool with variable sensitivity and specificity, a false positive result may occur due to contamination of amyloid deposits by serum proteins.^34^^,^^35^ On the other hand, a false negative result can be due to epitope loss during tissue fixation or by low antibody affinity for cross beta fibrils structure.^9^^,^^34^^,^^36^^,^^37^ Nowadays, LD/MS is considered the gold standard in amyloid typing, which is essential to guide the therapeutic option.^37^ By this technique, diagnosis, and subtyping of amyloidosis are based on the presence of large spectra of the amyloidogenic precursor protein and other signature proteins that occur in all amyloid types, such as apolipoprotein E, apolipoprotein A IV, and serum amyloid P component.^19^^,^^35^

The therapeutic approach in our case should also be discussed. The international therapeutic consensus was followed since the diagnosis of DADS in the absence of hematological neoplasia had been made.^2^^,^^7^ In this setting, corticosteroids, intravenous immunoglobulin (IgIV), or plasma exchange (PE) are equally effective as first-line options, and immunosuppressive agents could be used in case of non-responsiveness.^7^ IgIV and PE were not used due to restrictions to CIDP in our public health system and the presence of severe dysautonomia, respectively. As another therapeutic option in our service and before being evaluated by a hematologist, Fludarabin was introduced due to response failure to MP associated with Cyclophosphamide. When TTR mutation was confirmed, the patient had no longer clinical indication for Tafamidis, the only drug available in our public health system, or liver transplantation.

In addition to the complexity of diagnosing and subtyping amyloidosis, some limitations of our tertiary public service might have contributed to the delay in the diagnosis, such as non-refunding for genetic testing, the absence of a routine immunohistochemical panel for identification of the three most common types of amyloid (AL, ATTR, and AA), and delay in scheduling medical appointments with specialists. Also, this case report highlights the importance of more advanced techniques in typing amyloid deposits.

## CONCLUSION

Sometimes misdiagnosing a condition does not result from medical unawareness of the disease. The presence of overlapping clinical manifestations, delay in the appearance of characteristic signs of the disorder, combined with unavailability of specific diagnostic tools, and difficulties in obtaining an expert opinion may overall explain the occurrence of misdiagnosis, especially in the context of rare diseases. Establishing a reference center dedicated to amyloidosis with the availability of more advanced techniques in its subtyping can improve diagnostic procedures, reduce the time from symptoms onset to diagnosis and allow the institution of appropriate therapy, preventing harmful procedures and organ deterioration.
